# The Effect of Climate Change on Emergence and Evolution of Zoonotic Diseases in Asia

**DOI:** 10.1111/zph.70007

**Published:** 2025-09-01

**Authors:** Roger S. Morris, Masako Wada

**Affiliations:** ^1^ Massey University, EpiCentre, School of Veterinary Science Massey University Palmerston North New Zealand

**Keywords:** Asia, climate change, disease emergence, disease evolution, epitype, Zoonoses

## Abstract

As the climate of Asia changes under the influence of global warming, the incidence and spatial distribution of known zoonoses will evolve, and new zoonoses are expected to emerge as a result of greater exposure to organisms which currently occur only in wildlife. In order to evaluate the risks attached to different transmission methods and organism maintenance mechanisms, a classification system is provided which allocates diseases into nine epitypes. All animal diseases and zoonoses recognised as globally important can be categorised into an epitype, or in a few cases more than one epidemiologically distinct epitype. Within each epitype, evidence available on the effects of climatic factors is provided for selected diseases of zoonotic importance to illustrate likely future evolution of these diseases and the extent of currently available evidence for different diseases. Factors which are likely to influence the emergence of novel zoonotic pathogens in Asia are outlined. The range of methods available for analysis, prediction, and evaluation of likely changes in disease occurrence under the influence of climate change has grown rapidly; an introduction is given to the types of tools now available. These methods will need to be integrated into a surveillance and response strategy for Asia, and an approach to achieve this is outlined.

**Summary**

- Zoonotic diseases have been grouped into nine categories termed epitypes, each of which differs in transmission routes and maintenance methods in host populations and/or environments.
- Climate change influences each epitype in different ways. As a result, the diseases within a particular epitype have probabilities of emerging and/or evolving to display new epidemiological characteristics which differ from those in other epitypes.
- The nature and strength of climate effects on each epitype help predict future emergence of new zoonoses and evolution of existing diseases; hence, they reveal likely effects on the relative importance of different zoonoses, with particular reference to Asia.

## 1 Future Climatic Trends in Asia

The next 20 years will see a complex series of changes in the climatic factors which influence disease epidemiology. An overview of predicted changes in the climate of Asia is provided in the Sixth Assessment Report of the Intergovernmental Panel on Climate Change (Shaw et al. 2022). This report expects that rising temperatures will increase the likelihood of heatwaves across Asia, droughts in arid and semi‐arid areas of West, Central and South Asia, delays and weakening of the monsoon circulation in South Asia, floods in monsoon regions in South, South East and East Asia, and melting of glaciers in the Hindu Kush Himalaya region.

These broad scale predictions can be supplemented with more detailed country‐specific predictions. Within each country, different climate models may project varying climatic trends across localities (Lawler et al. 2006), shaped by a complex interplay of factors. At higher resolution, multiple variables interact dynamically, resulting in highly localised and diverse abiotic conditions, making it challenging to generalise future trends. Additionally, human activities, including greenhouse gas emission levels and shifts in land use, may further influence climatic outcomes, adding layers of uncertainty to future projections. Each country would require a separate evaluation of likely trends, which is outside the scope of this paper.

As an example, Bangladesh has been studied extensively and is likely to be one of the more severely affected countries; so it is used to illustrate how more localised expectations can be developed. Using a hierarchical clustering approach (Mahmud et al. 2022) the country can be divided into two main climatic regions. One cluster is located in the northern part of the country that includes drought‐prone and vulnerable regions, whereas the second cluster contains rain‐prone and hilly regions that are found mostly in the southern part. This allows assessments to distinguish between different climatic regions within the country.

Temperature levels have been increasing steadily over the last 50 years with a recent increase in the upward trend (Rahman and Lateh 2017); extreme temperature events have become more common (Mallick et al. 2022; Choi et al. 2021). Monsoon precipitation is decreasing and shifting in time, increasing the expected frequency of both floods and droughts (Mohsenipour et al. 2020; Khan, Islam, Das, et al. 2020), and shifting the spatial distribution of effects (Al Mamun et al. 2024). These adverse trends are predicted to continue (Khan, Islam, Bala, and Islam 2020).

A range of downscaling methods have been used to provide finer scale spatiotemporal assessments of climatic patterns (Alamgir et al. 2019; Ishaque et al. 2021), and methods of minimising biases in these assessments have been developed (Mosier et al. 2018). At a downscaled level within Bangladesh, satellite data has been used to measure land surface temperatures. This data can be used to deduce likely effects of changes over the last decade on agricultural productivity, and to predict future trends (Kafy et al. 2021).

Therefore, despite the inherent uncertainty and potential bias in climate projections, it is possible to provide climate predictions at different spatial scales in order to understand the effects of these predictions on likely future trends in disease occurrence.

## 2 Consequences for Asian Ecosystems

Climate change will have a wide range of effects on ecosystems—both natural‐ and human‐managed (Pandit and Sharma 2023). This in turn will have a range of effects on animal populations, and hence on the emergence and evolution of zoonoses, to be discussed in detail below. One of the ways in which ecosystems are affected is through reduction in ecosystem services—the benefits which the human population receives from natural ecosystems. These are divided into provisioning, regulating, supporting/habitat, and cultural services, all of which are being affected in Asia (Dang et al. 2021). Changes in land use and landcover are progressively occurring, and will have substantial effects on both ecosystem services (Gomes et al. 2021) and biodiversity (Hughes 2017). Asia has many middle‐to low‐income countries, where maintaining ecosystems often takes lower priority compared with other socioeconomic goals, such as economic development and poverty alleviation. Hence substantial adverse ecosystem changes are happening in Asia as their economies grow. In Bangladesh, a study predicted that these changes may cause serious damage to the biomass, water availability, and the genetic material from all biota (Hoque et al. 2020). Climate change has both physical and biological impacts on ecosystem function, with consequences for animals and vegetation (Kilroy 2015). This in turn can affect the size and location of the distribution ranges of mammalian species (Markov et al. 2022) and birds (Moller 2013), with implications for disease spread, and hence for both animal and human health (Myers et al. 2013). Policy responses to all these changes in Asian countries have been quite limited (Dang et al. 2021), in part because limited financial and institutional resources make it challenging to implement effective policies that preserve natural habitats and biodiversity.

## 3 Epidemiological Factors Influencing Emergence and Evolution of Zoonoses Under Climatic Changes

A range of factors can influence how climatic changes affect the occurrence and spatial distribution of different types of diseases.

Each organism—from viruses to mosquitoes to elephants—has a thermal performance curve, which represents its capacity to perform its normal functions as temperature changes (Molnar et al. 2017; Rezende and Bozinovic 2019). It has a minimum temperature below which it cannot function and may die or become inactive, an optimum temperature at which it performs most effectively, and a maximum temperature above which it cannot function and may die or become inactive. While this concept has been primarily applied to ectotherms, it is applicable in principle to all organisms (Levesque and Marshall 2021) and is directly relevant to the overall effects of climate change (Fox 2018).

As mean annual or seasonal temperatures change, and extreme temperature events occur more frequently due to medium‐ or long‐term influences, pathogen survival in the environment or ease of transmission can change (Rosenberg et al. 2018; Zhang et al. 2010; Akil et al. 2014). Not only vector‐borne diseases (Franklinos et al. 2022) but enteric diseases can show seasonality due to the interaction of multiple factors (Lal et al. 2012; Djennad et al. 2019). Satellite‐derived meteorological data can be used to predict future weather conditions conducive to rotavirus outbreaks (Hasan et al. 2018). The geographical range of vectors and hosts may also change (Furlong et al. 2023; Brinkhoff 2022), as may their susceptibility to infection with the pathogen or parasite. Short term temperature changes—such as heat waves (Milazzo et al. 2017, 2016) or periods of unusually cold weather (Burkart and Kinney 2017)—can alter local survival of organisms and cause sharp increases or reductions in disease incidence due to alterations in the transmissibility of pathogens and parasites.

Rainfall level and temporal pattern can directly affect pathogen and parasite availability and survival, and can alter the frequency of human exposure to them. Floods, droughts and other extreme weather events can affect the same epidemiological processes, but in a more severe form. The Sixth IPCC Report (Shaw et al. 2022) predicts an increased likelihood of unfamiliar, unusual or unprecedented wet and dry events worldwide. Oscillation patterns also suggest a higher probability of experiencing extreme cyclones, typhoons and hurricanes. Heavy rainfall days are linked to increased childhood diarrhoea (Wu et al. 2014), as are flood events (Hashizume et al. 2008). Temperature and humidity can interact to enhance disease transmission under Asian conditions (Hridoy et al. 2021; Islam et al. 2021).

In addition, climatic conditions comprise numerous interacting variables, requiring a multidimensional approach to understand their impacts on diseases. Temperature and moisture level interact to produce humidity levels, which affect pathogen transmissibility for airborne pathogens. It has been suggested that increasing climatic variability will result in pathogens evolving reduced sensitivity to climate fluctuations (Koelle et al. 2005); and hence moderate the impact of climate change.

Vector‐borne diseases are influenced by the effect of climate on their vectors, but also by the ecology of the environment which ticks (Ogawa et al. 2024), snail intermediate hosts (Yang et al. 2005) and insects (Le Flohic et al. 2013) inhabit.

As well as direct effects on diseases, climate change can induce large‐scale migration, which can then affect the health of both migrants and resident populations by changing disease patterns (Hunter et al. 2021).

The influence of these factors will now be considered for specific categories of diseases.

## 4 Epidemiological Classification of Diseases Into Epitypes

In order to categorise animal diseases and zoonoses into groupings which share their main characteristics in common, including the influence of climatic factors, the concept of classifying diseases into epitypes is introduced here. An epitype can be defined as a description of the main epidemiological characteristics of diseases which allow a pathogen to be maintained and disseminated in a host population. In this context, maintenance means the mechanisms by which the pathogen persists in the animal population from which zoonotic infection arises and/or in the environment, and where appropriate also in the human population. The term epitype was coined by the lead author many years ago for this specific purpose. The same word is used in very different contexts in botany (Turland et al. 2017) and epigenetics (Meagher 2010), but there is no overlap in meaning.

This enables epidemiologically similar diseases to be grouped together for the purposes of analysis, and in this case for prediction of the effects of climate change. There are nine epitypes in this classification. It is considered that all diseases fit into an epitype, although a small proportion of diseases have at least two quite distinct transmission mechanisms, and therefore must be included in more than one epitype.

- Epitype 1—directly transmitted from host to host, with maintenance entirely in hosts (including sexual and vertical transmission).
- Epitype 2—directly transmitted from host to host, with maintenance principally in hosts—but substantial environmental influence on frequency of transmission for some agents.
- Epitype 3—transmission mainly reliant on fomites as intermediates in transmission, maintenance in hosts.
- Epitype 4—transmission through food/feed or consumed water, long‐term maintenance in hosts and short‐term maintenance in environment.
- Epitype 5—transmission by mechanical vector, maintenance in hosts.
- Epitype 6—transmission by facultative biological vector, the vector phase does not involve a different replication phase for the agent, maintenance in hosts.
- Epitype 7—transmission through an obligatory cycle involving two different vertebrate species, maintenance shared between two types of hosts.
- Epitype 8—transmission through an obligatory cycle involving an invertebrate host in which the pathogen must go through a specific development stage before it can infect a vertebrate host. Maintenance shared between two types of hosts.
- Epitype 9—transmission through a nonanimal biotic reservoir or through the abiotic environment, such as water or soil, no host‐to‐host transmission, maintenance entirely in environment.

To provide some insight to the numbers of diseases within each epitype, all 120 diseases of mammals and birds which are currently or have been recently in the World Organisation for Animal Health (WOAH) list of reportable diseases were evaluated to put them into the nine epitypes. The categorisation was produced by researching the epidemiology of each disease on the WOAH list, and classifying it into one, or in nine cases multiple epitypes.

There were no diseases which were unable to be categorised. Six diseases have two epidemiologically distinct transmission mechanisms and three have three mechanisms. These diseases were therefore included in more than one epitype, whereas 111 could be categorised into a single epitype. The count of epitypes is shown in Table 1. There are diseases in all nine epitypes. Forty six of the 120 diseases are zoonoses.

**TABLE 1:** Number of diseases categorised in each epitype in the WOAH list of animal diseases (*n* = 120) and the author‐curated list of important zoonoses (*n* = 41). Diseases are counted multiple times if they fit into multiple epitypes.

Epitype	WOAH animal disease list	Animal diseases including zoonoses	Zoonoses in people
1	12 (9%)	13 (7%)	7 (7%)
2	70 (53%)	92 (52%)	38 (37%)
3	8 (6%)	10 (6%)	10 (10%)
4	1 (1%)	10 (6%)	16 (16%)
5	4 (3%)	5 (3%)	3 (3%)
6	24 (18%)	30 (17%)	18 (18%)
7	4 (3%)	4 (2%)	4 (4%)
8	5 (4%)	6 (3%)	2 (2%)
9	4 (3%)	7 (4%)	3 (3%)
Total	132	177	101

There is no comparable internationally agreed official list of zoonoses of importance. An additional list of 41 zoonoses was therefore produced from lists provided by the United States Centers for Disease Control and Prevention (Anon 2024), the British government (Anon 2019) and diseases considered in this paper. This is not a comprehensive list of all known and suspected zoonoses, but it represents the range of zoonoses considered to be globally important.

Fourteen of these diseases have two transmission mechanisms so fit into two epitypes. Hence there are 101 epitypes for the 87 diseases. The lists of diseases classified by epitype are in Table 1. The list of zoonoses has a higher proportion of Epitype 4 diseases (16%) than the animal disease list (6%) and lower proportion of Epitype 2 diseases (37%) than the animal disease list (52%) because foodborne zoonoses are more important in people than in animals, but overall the lists have similar proportions of epitypes. The full list of diseases and their epitypes for transmission between animals and transmission to people for zoonoses is provided in Supporting Information.

## 5 Climatic Influences on Different Disease Epitypes

Diseases which are grouped together in a single epitype share similar influences of climatic factors because they have similar transmission mechanisms and maintenance mechanisms in populations. Hence, at an overview level, they can be considered as a group; then, where necessary, individual differences within the epitype can be considered.

For each epitype, specific zoonotic examples have been selected to illustrate the range of influence of climate change on diseases within the type. The amount of evidence for each disease is also covered, since there are very variable amounts of evidence for different diseases.

### 5.1 Climatic Influences on Epitype 1 Diseases

Diseases directly transmitted from host to host, with maintenance entirely in hosts (including sexual and vertical transmission).

In principle, these are the diseases least likely to be directly influenced by changes in climatic factors, yet climatic influences on their occurrence have been reported. Rabies is not expected from first principles to be directly influenced by climate change in Asia because the virus is transmitted entirely by bite wounds, mainly from dogs. Asia has a particularly high incidence of human rabies, especially in India (Hampson et al. 2015) and China (Guo et al. 2018). Studies in various parts of Asia have found that the incidence of human rabies is associated with higher ambient temperatures (Guo et al. 2018; Naveenkumar et al. 2022; Lachica et al. 2020; Ahmed et al. 2019). Behaviour patterns and the density of dog and human populations may be influential, but the causal mechanism has not been investigated. It is expected that climate change may cause a small direct increase in human rabies, but this may be exacerbated by indirect consequences of climate change on hosts.

Trichinellosis is transmitted by consumption of muscle from an infected animal. Globally it has moved from being principally due to consumption of infected domestic pigmeat to being more a wildlife‐associated disease due to consumption of a variety of ‘bushmeat’, although in Asia both domestic pigmeat and wild boar are important sources (Yera et al. 2022). The earlier classification into the single species *Trichinella spiralis* has been replaced by a complex of species, each with different geographical distributions and climatic influences (Pozio and Zarlenga 2013). Maintenance of infection in animal populations is predominantly due to consumption of carrion, and survival of parasites in muscle of dead animals is favoured by lower temperatures (Tolnai et al. 2014). However, increasing consumption of meat from a wide range of wildlife species due to population pressure in Asia will increase human exposure (Greatorex et al. 2016). Hence, climate change is likely to have different effects on incidence of human trichinellosis according to location, increasing in some regions and decreasing in others.

*Toxoplasma gondii* is one of the organisms which fits in more than one epitype. While sexual reproduction occurs only in felids, the definitive hosts, asexual reproduction occurs in a wide variety of species. It belongs to Epitype 1 because vertical transmission occurs in the human population between the mother and unborn child, producing severe effects in many cases of congenital toxoplasmosis (Singh 2016). There is also evidence suggestive of sexual transmission in people (Singh 2016). The disease appears to be sensitive to a number of climatic influences, including temperature, precipitation, and relative humidity, when assessed between different locations (Singh 2016; Rostami et al. 2021), suggested to be related to interaction between survival time of oocysts and human behaviour patterns. Areas of the world with higher ambient temperatures have a higher incidence of congenital human toxoplasmosis (Rostami et al. 2019), especially areas with high humidity (Singh 2016). Vertical transmission is considered likely to also occur in a range of other species (Thompson 2013). There is geographical variation in the locations of different Asian genetic strains of *T. gondii* which may influence their ecological behaviour and climatic sensitivity, but this has not yet been determined (Chaichan et al. 2017). Overall, climate change is expected to increase human toxoplasmosis by becoming more prevalent in warm and humid areas that favour the maintenance of *Toxoplasma gondii* in the environment.

Because transmission requires direct contact between hosts, as yet undetected animal disease agents in this epitype are less likely to emerge as a result of climate change than those in other epitypes.

### 5.2 Climatic Influences on Epitype 2 Diseases

Diseases directly transmitted from host to host, with maintenance principally in hosts—but with substantial environmental influence on frequency of transmission for some agents.

Diseases in this epitype are sensitive to environmental influences of various types, both short term and long term.

Avian influenza is of substantial global concern because of geographical expansion and emergence of strains with a wider host range. It will be affected in complex ways by climate change. Environmental persistence of the virus is much longer at low ambient temperatures (Dalziel et al. 2016; Brown et al. 2009); so rising temperatures will reduce the length of time virus is available in the environment, particularly in the breeding sites of reservoir birds. Influenza viruses can be transmitted by fomites, droplets, and aerosols, with small particle aerosols being most important. Transmission by aerosol is more effective at lower temperatures (Wang, Prather, et al. 2021). However, the global ecology of avian influenza is strongly influenced by bird migration patterns, and these are likely to change as a result of rising temperatures (Prosser et al. 2023; Morin et al. 2018). Temperature and precipitation influence the number of risky birds in particular locations at specific times (Arikawa et al. 2019), and the mixing of different species can influence the evolution of new influenza strains (Blagodatski et al. 2021). Hence, the influence of climate change on avian influenza is likely to be particularly complex, and likely to reduce the importance of the disease in some locations and increase in others, including spread to previously uninfected host species.

Leptospirosis is largely a water‐borne infection derived from wildlife and domestic animals. Increasing ambient temperatures will increase the occurrence (Ehelepola et al. 2019; Warnasekara et al. 2021; Baharom et al. 2021) of the disease. This is not only due to increased survival of the organism in water and soil (Bierque et al. 2020; Cucchi et al. 2019), but also due to the fact that at higher water temperatures people undertake water activities that increase their exposure. Increased precipitation is also linked to subsequent incidence peaks of the disease in people (Ehelepola et al. 2019; Warnasekara et al. 2021; Rees et al. 2023; Convertino et al. 2021), and flooding following intense rainfall is particularly important (Romero et al. 2021; Tabucanon et al. 2021; Naing et al. 2019). As a result of these factors, the spatial distribution and incidence of leptospirosis in areas of higher precipitation are expected to increase significantly (Warnasekara et al. 2021; Dhewantara et al. 2022; Luenam and Puttanapong 2020; Douchet et al. 2022). Because of the wide range of host species and their changing distributions, the disease is expected to grow in importance in urban environments (Lau et al. 2010; Andersen‐Ranberg et al. 2016).

Nipah virus is known to occur in eight bat species across nine countries in Asia from India to the Philippines, with separate clades in the west centred on Bangladesh and the east on Malaysia and its neighbours (Sun et al. 2024). Human infection occurs when bats contaminate human food (such as palm sap) or infect an amplifying species (pigs in Malaysia). Climate effects on the incidence of Nipah virus depend directly on seasonal (Cortes et al. 2018) and long‐term changes in temperature (Sun et al. 2024), precipitation and weather patterns (Latinne and Morand 2022), but also indirectly through effects on bat density and ecology (McKee et al. 2021). The interaction of these effects is expected to increase the spatial distribution of spillover events in future (Sun et al. 2024), with variations between regions due to ecological factors (Chaiyes et al. 2022).

Epitype 2 is the most common transmission method for both animal diseases and zoonoses, and is likely to be the most common epitype of new diseases which emerge in future as important human pathogens. In Asia, these emergent diseases are likely to be transferred from the reservoir host to an intermediate susceptible species known as a bridge host (Caron et al. 2015), which has closer contact with people and occasional contact with the reservoir host. Then, as an exceptional event, infection is transferred to susceptible people. Transfer of both SARS‐CoV‐1 and SARS‐CoV‐2 to the human population occurred by this indirect pathway from bat reservoir hosts. While the role of a range of bat species as reservoir hosts for emergent viruses is now well recognised, other wild animals are also likely to be the reservoirs for future emergent organisms of Epitype 2.

There are recent examples of the complex ways in which Epitype 2 diseases can evolve to produce new epidemiological patterns. Avian influenza H5N1 is transmitted within and between most species mainly by the respiratory route. Infection of people remains relatively rare, and direct transmission between people by this route has not yet been proven. When human cases occur, the affected people do not excrete sufficient virus by the respiratory route to maintain continuing direct human‐to‐human spread. A relatively small change in the viral genome would potentially increase the transmissibility of the virus by airborne routes, and this evolution could lead to a substantial epidemic or pandemic. Fortunately, this has not so far occurred, but continuing spread of the virus in a range of animals on a large scale could allow a strain to emerge that is highly contagious between people.

Human behaviour patterns may also influence the ways in which new types of zoonotic outbreaks can arise due to Epitype 2 agents. Ebola virus disease has been recognised in Africa since 1976, but outbreaks had been small, associated with animal contact and rural environments until the West African epidemic of 2014–2016. Early cases in that epidemic moved from rural to urban areas in search of better health care, including across national borders. Due to funeral practices in the affected countries, direct transfer of infection between people became the dominant transmission route, resulting in a large urban outbreak in the three affected countries and spread to seven additional countries due to movement of infected people (Alexander et al. 2015).

Recent experience with avian influenza and coronaviruses has illustrated the capacity of Epitype 2 disease agents that are transmitted by the airborne route to undergo genetic evolution during the process of establishment in the human population. This is particularly significant for RNA viruses (Williams et al. 2021), which have an imperfect replication method that results in continuous production of variant genotypes of virus, a small proportion of which show higher Darwinian fitness. They can then become the dominant strain circulating in the human population if the virus has a high transmission rate (R_0_) between people—as occurred in the COVID‐19 pandemic. This enhances the capacity of RNA viruses such as influenza A and SARS‐CoV‐2 to cause epidemics and pandemics.

There is a risk that Epitype 2 organisms circulating in animals, such as currently undetected RNA viruses, may enter the human population and evolve to cause major disease outbreaks (Zhang et al. 2020; Woolhouse and Brierley 2018). Epitype 2 agents are the ones most likely to emerge as new diseases.

### 5.3 Climatic Influences on Epitype 3 Diseases

Disease transmission mainly relies on fomites as intermediates in transmission, maintenance in hosts.

Attachment to fomites provides enhanced survival of pathogens in this type; they are quite sensitive to short‐term climatic influences, as well as benefitting from longer‐term climatic changes.

*Salmonella* species can be directly transmitted, but these organisms are persistent in the environment and can therefore be transmitted on a range of fomites, including water. As short‐term ambient temperature increases, there is greater environmental persistence (Hellberg and Chu 2016) and human case numbers increase in both tropical (Wang et al. 2018; Aik et al. 2018) and more temperate climates (Zhang et al. 2010; Robinson et al. 2022; Lal et al. 2016). Periods of extreme heat are associated with increased case numbers (Milazzo et al. 2016; Morgado et al. 2021). Periods of higher than usual rainfall and high humidity are also associated with increased cases (Zhang et al. 2010; Wang et al. 2018; Aik et al. 2018). Thus, changes in climate in some areas are likely to be associated with increased environment‐derived salmonellosis.

There are multiple *Brucella* species, each with a host range of terrestrial or aquatic animals. The most important are *Brucella melitensis* , principally in sheep and goats, and *Brucella abortus* in cattle and other domestic and wild ruminants. The main route of transmission between animals is through environmental contamination by placentas and other fomites from aborting or parturient animals. Transmission to people is by contact with these fomites or consumption of unpasteurised dairy products. Although *Brucella* species are intracellular parasites, they are readily transmitted on fomites, and the diseases are globally important (Dawood et al. 2023). Transmission is more effective at lower temperatures (Aune et al. 2011), and the spatial distribution of human cases favours areas with lower temperatures and higher relative humidity (Zhang et al. 2024; Faramarzi et al. 2019). Therefore, brucellosis is one of the zoonoses which may decline in importance as temperatures rise in currently affected areas.

Epitype 3 disease agents are capable of emerging and causing disease outbreaks, but it is generally easier to control organisms that are spread on fomites rather than by airborne routes. Hence, they are more likely to modify the prevalence of endemic disease in particular at‐risk populations rather than cause epidemics in wider populations.

### 5.4 Climatic Influences on Epitype 4 Diseases

Diseases transmitted through food/feed or consumed water, long‐term maintenance in hosts and short‐term maintenance in environment.

These diseases vary considerably in susceptibility to climatic influences; some are particularly prone to cause outbreaks as a result of extreme weather events.

*Campylobacter* species are thermophilic—and therefore increasing temperatures will favour their role as human pathogens, but they are microaerophilic and use adaptation mechanisms to survive at normal oxygen levels (Gölz et al. 2018; Bronowski et al. 2014). Although they are very sensitive to adverse environmental conditions, these adaptation mechanisms allow them to be a major cause of gastroenteritis (Li et al. 2019). Domestic and wild animals act as reservoirs of infection, without clinical manifestations of disease. Most human infections occur due to consumption of a wide range of food items which become contaminated with the organisms (Gölz et al. 2018); but large waterborne outbreaks occur when drinking water supplies become contaminated with animal faeces (Gilpin et al. 2020). Elevated temperatures are linked to subsequent occurrence of campylobacteriosis (Rosenberg et al. 2018; Djennad et al. 2019; Lake et al. 2009). Heavy rainfall events can precipitate outbreaks, such as by causing contamination of drinking water supplies (Gilpin et al. 2020; Soneja et al. 2016). These factors can combine to cause seasonal peaks of cases (Lal et al. 2012; Djennad et al. 2019).

The genus *Cryptosporidium* includes a range of protozoon species which cause human disease through contamination of food and water (Ryan et al. 2014; Golomazou et al. 2024; Xiao and Feng 2008). Cryptosporidiosis is an important health issue in Asia (Lim et al. 2010), especially in young children (Khalil et al. 2018). Survival of oocysts is adversely affected by higher temperatures, both in terrestrial environments of different kinds (Zuo et al. 2023; Meng et al. 2021; Wang et al. 2023) and in water (King et al. 2005). Elevated precipitation was associated with subsequent increased prevalence of *Cryptosporidium* (Grembi et al. 2024). Severe weather events, especially flooding, have been a precipitating factor in large outbreaks of cryptosporidiosis (Poglayen et al. 2023; Grout et al. 2024). Prediction of the expected trend in cryptosporidiosis is complicated by human behaviour factors. While oocysts have shorter survival at higher temperatures, people tend to expose themselves to water bodies likely to contain oocysts when the weather is warm. It has been predicted that case numbers of cryptosporidiosis will rise sharply in the future (Chua et al. 2021), but the epidemiological evidence base for this prediction is very limited. This is one of the more difficult diseases to predict with any confidence.

*Toxoplasma gondii* is a member of the Phylum Apicomplexa, all obligate intracellular protozoan parasites. It has multiple transmission mechanisms; hence, it has already been described in Epitype 1 because of its vertical transmission capability. It also clearly qualifies for inclusion in Epitype 4, as oocysts are ingested with food or water and can contaminate soil (Lelu et al. 2012; Kakakhel et al. 2021), pasture and crops (Atif et al. 2024). Felids are the definitive hosts in which sexual reproduction occurs, and oocysts are excreted in faeces. Virtually all mammals and birds can ingest the oocysts and become intermediate hosts (Wilson et al. 2024), but infection is inapparent in a high proportion of infected individuals. People become infected by consuming raw or undercooked meat or through direct contact with cat faeces. Human infection is very common, but clinical disease is limited to the circumstances described in Epitype 1. Clinical disease occurs in other species, particularly sheep, goats, deer and pigs, in the form of abortions, stillbirths and perinatal deaths (Stelzer et al. 2019). A fatal form of the disease occurs in some wildlife species. The effect of climate change on the occurrence of human toxoplasmosis was discussed in Epitype 1. Within the animal population, climate can affect oocyst survival in the environment (Lelu et al. 2012), spatial dissemination of oocysts and the ecology of definitive and transport hosts (Afonso et al. 2013), resulting in expanded geographical distribution of the parasite (Meerburg and Kijlstra 2009). Therefore, it is expected that *Toxoplasma* infection of animals will expand to new areas, so infection and disease in animals and hence in people will become more common under climate change.

It is likely that new Epitype 4 organisms, or particularly epidemiologically different variants of known organisms, will emerge from animal sources in the future. For most emerging pathogens which are Epitype 4 and have no other transmission mechanism, control should be possible by either removing the source or processing the animal feed, human food or water to remove the pathogen. However, Bovine Spongiform Encephalopathy (BSE) showed that this is not necessarily practical in all cases—especially for prion diseases. If a new prion disease were to arise, it may reach epidemic proportions before control could be initiated, because of the long incubation period and the inability to detect the disease prior to the development of clinical signs.

### 5.5 Climatic Influences on Epitype 5 Diseases

Diseases transmitted by mechanical vectors, maintenance in hosts.

Most of the diseases which fit this epitype are also included in other epitypes as well. If transmission occurs by mechanical vector, the organism is also likely to spread by one or more other modes as well, although screwworms are exceptions.

Old World screwworm fly (*Chrysoma bezziana*) and New World screwworm fly ( *Cochliomyia hominivorax* ) are zoonotic obligate parasites of mammals (including humans) during their larval stage. The adult fly deposits eggs into a skin lesion or body opening, which then develop through the larval stage by consuming body tissue and become free‐flying adults. In their different regions, they occur either side of the Equator, and the effect of climate warming would be to extend their realisable geographical range to higher latitudes, with fluctuating boundaries as minimum temperatures allow. This extension may be quite substantial for *C. bezziana* in Asia (Hosni et al. 2022, 2020; Zaidi et al. 2016; Wardhana et al. 2014) with increased risk of human cases (Zhou et al. 2019) and *C. hominivorax* in the Americas (Mulieri and Patitucci 2019). Unlike pathogens which depend for their local persistence on climatic conditions in each location, these flies can take advantage of whatever range expansion opportunities occur, both in the short and long term.

Mechanical vector transmission of other pathogens by insects is usually a secondary method of transmission of organisms which have other primary transmission mechanisms. Examples of diseases which are discussed within other epitypes include transmission of anthrax by tabanid and other flies in various regions (Anon 2008) and transmission of *Toxoplasma gondii* by cockroaches (Patel et al. 2022). A range of other zoonotic pathogens can be transmitted mechanically by a variety of insects (Khamesipour et al. 2018; Lin et al. 2024). Temperature increases are expected to increase both geographical range and numbers of these insects and therefore their transmission capacity; extreme weather events can add to these effects (Poglayen et al. 2023).

It is unlikely that Epitype 5 will be a significant source of new zoonoses.

### 5.6 Climatic Influences on Epitype 6 Diseases

Diseases transmitted by facultative biological vector, the vector phase does not involve a different replication phase for the agent, maintenance in hosts.

Japanese encephalitis is a mosquito‐borne flaviviral zoonosis which causes human disease and deaths across Asia (Moore 2021) and has recently shown major range expansion to become widespread in Australia due to unusual climatic conditions (Kwa et al. 2023). Other members of the flavivirus group have also shown capacity to take advantage of favourable climatic changes. There are five known genotypes of Japanese encephalitis virus, which differ in their geographical distribution and in some epidemiological characteristics, and they are part of a wider cluster of related zoonotic viruses (Mulvey et al. 2021). Ardeid birds are the principal maintenance hosts of the viruses, with an incompletely defined but substantial number of species involved (van den Hurk et al. 2009). Movement of these birds is a factor in extensions of the range of the disease. Bats can also act as maintenance hosts. Pigs are the main amplifying hosts leading to human exposure, but wildlife species may contribute in some locations (Morris and Bingham 2023). Transmission of the virus to humans is by mosquitoes, and nearly 30 species in several genera are either confirmed or suspected vectors, each with its own climatic range determinants and ability to expand into new areas (Auerswald et al. 2021; Van den Eynde et al. 2022). This virus can also spread directly between pigs without involvement of mosquitoes (Ricklin et al. 2016).

Climate change is expected to expand the distribution of Japanese encephalitis virus (Pearce et al. 2018) and increase incidence rates of human disease (Hsu et al. 2008), since it will be reaching naïve populations. The changes will be complex, since they depend on the composition and size of the populations of multiple mosquito species, changes in distribution and ecology of Ardeid birds and density of amplifying species, especially pigs. The direct effects of climate change on the virus will be minimal, but the indirect effects via both insect and vertebrate hosts will be considerable. For vector mosquitoes, temperature increase is expected to raise vectorial effectiveness; although the results vary between vector species and can be quite complex within a species (Ciota et al. 2014). Temperature and precipitation can interact to influence mosquito numbers (Murty et al. 2010) and hence Japanese encephalitis incidence in the human population (Bi et al. 2003). The impact of these factors can be modified by the land use pattern in the area (Le Flohic et al. 2013; Hunt et al. 2017).

Because of its severity in humans and its capacity to spread rapidly into new areas as a result of movement of host species and various vector mosquito species, Japanese encephalitis is expected to attract considerable regional attention in future years as climate change continues. Although only a small proportion of infected people develop severe disease, children are particularly at risk of the disease.

Crimean Congo Haemorrhagic Fever (CCHF) is also expected to expand its geographical distribution and impact on human health in Asia as climate change occurs, although the influential factors will operate in a different way. The reservoir of this virus is hard ticks, especially those of the genus *Hyalomma*. A wide range of vertebrates can be infected inapparently with the virus, with the main risk species being ungulates. Transmission of the virus to humans occurs principally through tick bites. However, direct spread from animals to people also occurs through contact with blood during slaughter and other procedures undertaken with animals, while significant nosocomial transmission can occur when affected people are being treated.

The epidemiology of the CCHF virus and its relationship to ticks and vertebrate hosts is complex (Kuehnert et al. 2021). While a variety of hard ticks can become infected, it is uncertain which ones are competent vectors, outside the genus *Hyalomma*. It might appear that infection could only expand slowly, through a range of mammals and birds carrying infected ticks to nearby locations, with the infection establishing and maintaining only if the tick species can populate the area. However, the ticks are carried by a range of migratory birds, and therefore range expansion can occur quite rapidly (Estrada‐Pena et al. 2021; Jameson et al. 2012).

Therefore, climate change is expected to enable infected tick populations to spread into new areas, and to increase in some endemic areas. Both higher temperatures and humidity are necessary for persistent tick infection (Gargili et al. 2017) and an association has been found with the occurrence of human cases (Yilmaz et al. 2023; Nili et al. 2020). Land use patterns and habitat fragmentation can also affect the epidemiological situation of CCHF (Estrada‐Pena et al. 2010). It is possible to predict ecological niches suited to the establishment of the disease as climatic factors and land use change (Hekimoglu et al. 2023; Yang et al. 2021; Gale et al. 2010).

Scrub typhus is a disease caused by the intracellular Gram‐negative Alphaproteobacterium *Orientia tsutsugamushi*. Various synanthropic species of rats and mice are the main reservoirs responsible for human infections, but over 100 mammal species have been shown to carry infection (Elliott et al. 2019). Transmission occurs when trombiculid mites resident on reservoir species bite people. The disease occurs widely across Asia and has been expanding its distribution in recent times, often due to the adoption of new farming practices (Zangpo et al. 2023) and consequent habitat modification (Elliott et al. 2019; Zangpo et al. 2018). Climatic factors influence the occurrence and seasonality of the human disease across Asia from India (D'Cruz et al. 2024) to Japan (Seto et al. 2017) and South Korea (Chang et al. 2024), with higher temperatures (Qian et al. 2024; Han et al. 2023) and higher relative humidity (He et al. 2022; Bhopdhornangkul et al. 2021) typically linked to higher case numbers. The disease peaks in summer/autumn in more temperate regions (He et al. 2022; Bhopdhornangkul et al. 2021) and in the wet season in tropical regions (He et al. 2022; Bhopdhornangkul et al. 2021; Roberts et al. 2021). Because the mites are resident on the reservoir hosts—in contrast to tick infestations—the joint ecology of hosts and mites determines the epidemiology of the disease (Song et al. 2023; Chen et al. 2023). These various factors interact to produce the spatial distributions in different countries (Acharya et al. 2019; Ding et al. 2022) and influence extension to new areas (Zangpo et al. 2023).

Epitype 6 diseases are likely to evolve substantially under climate change. In addition, there are a substantial number of viruses in this epitype which have been identified in limited locations ‐ presumably due to local ecological factors ‐ and cause human infections (Wang, Wei, et al. 2021). They could potentially expand rapidly in distribution and disease impact if climatic circumstances changed, such as the arrival of a potentially more competent vector species in the local endemic area.

### 5.7 Climatic Influences on Epitype 7 Diseases

Diseases transmitted through an obligatory cycle involving two different vertebrate species, maintenance shared between two types of hosts.

The hydatid parasites which cause cystic hydatid disease (*Echinococcus granulosus sensu latu* and differentiated regional species within the group) and alveolar hydatid disease (*Echinococcus multilocularis*) both cause substantial disease in people (Woolsey and Miller 2021) across a wide range of countries (Deplazes et al. 2017). Alveolar hydatidosis is expanding in geographical distribution and importance in Asia, especially in China and central Asian countries (Woolsey and Miller 2021; Yin et al. 2023; Xu et al. 2023; Baumann et al. 2019). It is largely a sylvatic disease involving a range of wildlife species both as definitive and intermediate hosts. Ecological drivers of its distribution include lower temperatures and higher precipitation, but these interact with land cover and distribution of various host species in complex ways, in part because of the importance of over‐wintering of eggs in the environment (Simoncini and Massolo 2024). Expansion of the disease has been occurring in cooler areas of Asia where winter precipitation has increased. Consequently, this parasite is showing expansion of range and importance in different environments from diseases in other epitypes, due to the particular climatic changes occurring in these cooler regions (Atkinson et al. 2013).

Cystic hydatidosis caused by *Echinococcus granulosus* is mainly associated with domestic animals, with domestic dogs as the principal definitive host and farmed livestock of the particular area as the intermediate hosts. Incidence of human infection has increased progressively in Asia (Xu et al. 2023; Paternoster et al. 2021; Ghatee, Nikaein, et al. 2020), associated with changes in land use and increased scale of animal husbandry in the various regions, while combinations of temperature and precipitation patterns that favour longer egg survival in soil facilitate the spread of the parasite (Deplazes et al. 2017; Atkinson et al. 2013).

In contrast to directly contagious diseases such as those in Epitype 2, the two‐host cycle of these diseases, the complex interactions between numbers and activities of both definitive and intermediate hosts and the influences of climatic factors on egg survival time will result in focal distribution of areas in which there is expanded occurrence of these diseases, where several factors are favourable—such as in areas of higher elevation where the climate becomes milder.

Three tapeworm members of the *Taenia* genus are significant zoonotic parasites in this epitype—T. solium, T. asiatica (pork tapeworms) and *T. saginata* (beef tapeworm). Humans are the definitive host for the three tapeworms, while the intermediate hosts in which cysticerci occur are cattle for *T. saginata*, pigs for *T. solium* and predominantly pigs, but also cattle for *T. asiatica (*Mendlovic et al. 2021). The normal cycle involving adult tapeworms in people can be effectively managed and is not directly affected by climatic factors since it is caused by consumption of raw or undercooked meat or organ tissue containing cysticerci. However, there is an important aberrant pathway in which people consume eggs of *T. solium* (and possibly *T. asiatica* ) from a contaminated environment. In these cases, cysticerci can form in the central nervous system, causing the serious disease neurocysticercosis in humans (Garcia et al. 2014). This occurs across Asia (Wu et al. 2017) and is particularly important in countries where practices facilitate exposure to proglottids or eggs (Detha et al. 2022; Qian et al. 2020; Holt et al. 2016; Devleesschauwer et al. 2014). Infection risk of neurocysticercosis in humans is due to oral intake of eggs, which depends on factors such as scale of pig production, methods of human waste disposal and survival time of eggs in the external environment. Direct evidence is sparse and very dated (Gemmell 1978; Ilsee et al. 1990), but in an epidemiological study flooding was found to be an influence (Wardrop et al. 2015), and it has been proposed that increasing temperatures and longer dry periods will reduce egg survival in the environment (Pozio 2020). This is unsubstantiated by direct evidence, and is contradicted by evidence of the rising human caseload in Asia.

Diseases in this epitype are growing in importance due to the combination of demographic and land use factors, interacting with climatic influences. It is unlikely that any new diseases in this epitype will emerge; although new variants may be identified, as occurred with *T. asiatica* .

### 5.8 Climatic Influences on Epitype 8 Diseases

Diseases transmitted through an obligatory cycle involving an invertebrate host in which the pathogen must go through a specific development stage before it can infect a vertebrate host. Maintenance is shared between two types of hosts.

Leishmaniasis is a group of diseases caused by 53 species of the genus *Leishmania*, at least 20 of which infect humans. Almost all the significant pathogens are zoonotic, with the exception of *L. donovani* and *L. tropica* , although even these have been found in animals on occasion (Hong et al. 2020; Akter et al. 2016). *Leishmania* is an obligate two‐host protozoan parasite transmitted by at least 93 species of sandflies, in which obligatory replication occurs to produce the metacyclic promastigote, which is infectious for vertebrate hosts when bitten by the fly (Montaner‐Angoiti and Llobat 2023). These flies are members of the genus *Phlebotomus* in the Old World and *Lutzomyia* in the New World. There are three forms of the human disease caused by different species of *Leishmania*, some of which are zoonotic while others are anthroponotic. The maximum extent of geographical distribution of the diseases is determined by the range of each of the particular sandflies which transmit the *Leishmania* species (Pigott et al. 2014). Then within that range, the distribution is influenced by the range of mammalian hosts which are capable of transmitting the amastigotes into a blood meal for a suitable sandfly species. While the host may be a person for the zoonotic species, at least one and, in some cases, several animals may be reservoir hosts.

The distribution of the sandflies is determined by climatic conditions, and current changes in climate favour expansion of the ranges of many of the sandfly species (Koch et al. 2017). Because of the epidemiological complexity of the ways that *Leishmania* species, sandflies and reservoir hosts interact, it is necessary to consider specific situations rather than draw any general conclusions about the effect of climate on the human disease. This has been done best in Iran, in ways that are informative for the Asian epidemiological situation. The effects of climate and other factors in determining ecological niches for the *Phlebotomus* species have been examined (Sofizadeh et al. 2017; Cheghabalaki et al. 2019), while factors influencing expansion of the distribution of locally occurring *Phlebotomus* species have also been investigated (Ghatee, Fakhar, et al. 2020). Factors influencing the ecological niches suitable for specific reservoir species have also been assessed (Gholamrezaei et al. 2016). This information has been used to make various predictions of likely future expansion of zoonotic leishmaniasis in Iran (Shiravand et al. 2019; Abdolahnejad et al. 2021; Charrahy et al. 2022). Other countries will need to emulate this approach. Studies have been conducted on a more limited scale in China (Gao et al. 2018), Nepal (Dhimal et al. 2015) and Pakistan (Khan et al. 2021).

Fascioliasis in Asia is a two‐host disease in which the liver flukes *Fasciola hepatica* and *Fasciola gigantica* have their adult phase in a definitive mammalian host, including people. Eggs are excreted and produce miracidia which infect a range of species of Lymnaeid snails. Development in these intermediate hosts produces cercariae which become encysted on plant material as metacercariae. When these are consumed by a susceptible mammal, they develop into the adult form and complete the cycle. People become infected by consuming metacercariae with a variety of foods (Mas‐Coma et al. 2018). Infection occurs widely in Asia, in both animals and people (Rosas‐Hostos Infantes et al. 2023; Hoang Quang et al. 2024; Ahmed et al. 2024). In Vietnam, the number of human cases has increased dramatically in recent years (De et al. 2024).

Climatic factors influence each of the transmission stages of the flukes. The process of egg development, formation and hatching of miracidia and the proportion of eggs which produce miracidia are all favoured by elevated temperatures up to the point where unusually high temperatures inhibit the process (Modabbernia et al. 2024). Following penetration of miracidia into the snail, development of the sporocysts into redia results in the production and release of cercariae, which occurs much faster at higher ambient temperatures (Modabbernia et al. 2024). Metacercariae survive for extended periods at moderate temperatures, and survival at higher temperatures is enhanced by adequate humidity (Modabbernia et al. 2024; Meek and Morris 1979). Flooding (Poglayen et al. 2023) and drought can both modify local epidemiological outcomes.

Increasing temperatures will increase fecundity and growth of the intermediate host snails and hence population size, up to the point where optimal thermal range is exceeded; and snail survival is inhibited. The time taken for *Fasciola* larval development to be completed in the snail is reduced at higher ambient temperatures (Dube et al. 2023). Hence, it is expected that the prevalence and severity of human and animal fascioliasis will generally increase with climate warming.

Zoonotic babesiosis is an emerging zoonosis, first identified in 1957 and still with incompletely defined epidemiology. Various species of wildlife are reservoir hosts, and transmission is principally by ticks of the *Ixodes* genus. Nosocomial transmission also occurs by blood transfusion and organ transplantation. Human cases range from asymptomatic infection to severe disease and some deaths. Multiple species of *Babesia*, multiple species of ticks, and multiple wildlife hosts are involved in complex interrelationships (Young et al. 2019; Yabsley and Shock 2013). The disease has been diagnosed in several Asian countries so far, but not yet frequently. It is predicted that tick‐borne zoonoses will grow in importance with climate change (Estrada‐Pena et al. 2012; Gilbert 2021), but the epidemiological complexity of the syndrome and gaps in understanding make precise assessment of likely changes premature.

While the two‐host life cycles of these organisms would seem to make the emergence of new Epitype 8 diseases unlikely, the recent emergence of zoonotic babesiosis indicates that it may occur to a limited extent.

### 5.9 Climatic Influences on Epitype 9 Diseases

Diseases transmitted through a nonanimal biotic reservoir or through the abiotic environment, such as water or soil. Maintenance entirely in the environment.

The agents responsible for these diseases can persist in the environment for extended periods and typically cause animal and human infections opportunistically when exposure occurs. Some can also be directly transmitted between hosts. They are sometimes called sapronoses (Kuris et al. 2014) if the principal reservoir is in the physical environment (many disease‐causing fungi) or saprozoonoses if the organisms persist in the environment but also commonly infect animal hosts, which contribute to the quantity of organisms in the environment (anthrax). However, these terms are not in wide use.

Within this epitype, anthrax is important both globally and across Asia (Carlson et al. 2019). The distribution of human cases is closely tied to the distribution of animal cases, which in turn is localised to particular areas in the various Asian countries because of factors influencing long‐term survival of the organisms (Walker et al. 2022; Chen et al. 2016; Hassan et al. 2015; Islam et al. 2024). Soil characteristics are important in determining whether anthrax bacteria will persist in a particular location. Soil pH neutral to alkaline, adequate organic carbon and favourable cation exchange capacity were identified as supporting anthrax survival (Deka et al. 2022), consistent with earlier evidence that pH above 6.1 and high calcium concentration were favourable. It appears that there is not a soil replication phase; the spores simply survive for long periods in favourable soils (Hsieh and Stewart 2023). When the carcass of an animal which dies of anthrax decomposes, it raises soil nutrient levels and modifies the bacterial composition in the soil (Valseth et al. 2017). This enhances plant growth and makes the location attractive to grazing animals, especially in hot dry conditions when these sites tend to produce green patches of plants, which are grazed by animals that then become the next series of anthrax cases (Turner et al. 2014).

These factors interact with climatic conditions to produce the spatiotemporal pattern of anthrax cases in risk locations. In most areas where anthrax outbreaks occur, they are associated with hot, dry conditions for the reasons described above (Hugh‐Jones and Blackburn 2009). However, in Bangladesh, the outbreaks are associated with high temperature and rainfall in what is considered the anthrax season because these conditions favour access to anthrax spores in the husbandry system used for cattle (Hassan et al. 2015). In northern temperate areas of Asia, warming temperatures are associated with increased risk of anthrax outbreaks (Walsh et al. 2018) and in Siberia, melting of permafrost due to elevated temperatures produced a major anthrax outbreak (Ezhova et al. 2021). Therefore, anthrax is primarily controlled by environmental factors that allow persistence of anthrax spores in suitable soils for long periods. Exposure of animals is then precipitated by conditions which cause animals to gain access to the spores, with those conditions varying between different parts of Asia. Human cases are then due to exposure to affected animals or their products.

*Clostridioides* (formerly *Clostridium*) *difficile* is a spore‐forming anaerobe which has become an important emerging pathogen of the current century. It occurs widely in Asia (Usui 2020; Wu et al. 2022; Monaghan et al. 2022; Khun et al. 2023). It was identified in 1935 as a commensal of the lower digestive tract, but with the use of antibiotics from the 1950s, it became a significant cause of colitis and later other infectious processes in antibiotic‐treated people, as a nosocomial disease (Di Bella et al. 2024). Between 1999 and 2003, a new strain was detected in Canada which caused more severe disease and increased mortality (Pepin et al. 2004). This strain has since spread around the world and has substantially raised the importance of the organism. The organism has also become more important as a cause of infections in the community rather than just in hospitals (Kwon et al. 2017). Spores are very widely distributed in the environment and persist for extended periods (Jo et al. 2022; Esfandiari et al. 2021; Lim et al. 2020; Zhou et al. 2021).

Infection occurs in a wide range of domestic (Hernandez et al. 2020; Hain‐Saunders et al. 2022; Rupnik et al. 2024; Martin‐Burriel et al. 2017) and wild animals (Alam et al. 2019), with and without clinical signs (Silva et al. 2020), and some strains are shared between people and various animal species (Tsai et al. 2021; Redding et al. 2022; Werner et al. 2020). Transmission can occur between animals (Boarini‐Ferroni et al. 2020; Hopman et al. 2011). There is evidence that supports the view that infection is transferred from animals to people in some circumstances (Garza et al. 2023; Orden et al. 2017), but there is not yet comprehensive proof that animals act as reservoirs for human infection. There is surprisingly little research evidence on the role of climatic factors in the maintenance and transmission of infection. The evidence available so far is extremely fragmentary (Usui et al. 2017; Oh et al. 2021). Airborne dissemination of spores occurs both in hospitals (Best et al. 2010) and in animal facilities (Keessen et al. 2011), and the spores are widely found in foods (Bolton and Marcos 2023; Hazarika et al. 2023; Rodriguez‐Palacios et al. 2013).

*Clostridioides difficile* is growing considerably in importance as a human pathogen and also causes disease in animals in some cases. Its wide environmental distribution meets the requirements for Epitype 9, but possibly also for other epitypes, since the relative importance of different exposure and transmission mechanisms is not yet clear. Initially largely limited to nosocomial transmission, transmission within the community is becoming increasingly important. Whether animals play a significant part in this community spread remains to be clarified. The likely influence of climate change on the epidemiology of this organism remains to be resolved, due to a lack of sufficient evidence.

New organisms may emerge within this group due to environmental changes, such as occurred with *C. difficile* as described above; these changes may cause infection of both animals and people, with potential for exchange between the two as a zoonosis.

## 6 Analytical Methods

The nature of disease emergence and evolution typically means that many factors are directly and indirectly associated with the processes involved. Pathogens, hosts, vectors, transmission and maintenance mechanisms and environmental influences interact in complex ways which require analytical tools which dissect the contributions of the various factors. Availability of adequate and appropriate quantities of data is often a limiting factor on the precision with which the contribution of specific factors can be assessed.

A variety of analytical methods have been used to analyse the impacts of climate on diseases (N. R. Council 2001), and the range of tools is rapidly expanding. Only a few examples to illustrate the breadth of approaches can be mentioned here. These approaches aim to develop models which provide a better understanding of the relationships between climate and disease dynamics, using methods such as ecological niche modelling (Johnson et al. 2019; Escobar and Craft 2016; Liu et al. 2020). Increasing emphasis is being given to the development of methods to predict the potential impacts of climate change on disease emergence and evolution (Sadeghieh et al. 2020; Sargent et al. 2022; Woolhouse 2011). These models may provide spatial distribution maps (Chanda et al. 2023) or predict future spatiotemporal trends (Carlson et al. 2022; Tyrovolas et al. 2021; Ali et al. 2009). Time series analysis can also be used to identify temporal trends (Imai et al. 2015). Various methods of risk analysis can be used to assess the risk of introduction of pathogens into countries (Gao et al. 2022; Peeler et al. 2015; Condoleo et al. 2021; Cao et al. 2010). Social science methods are also being used to evaluate human behaviour and population dynamics on disease epidemiology (Li et al. 2023; do Thi Thanh et al. 2014). In order to make the range of analytical methods available to epidemiologists, decision support tools are being developed (Rocklov et al. 2023; Bingham et al. 2020).

The existing body of literature provides evidence on identified risk factors and qualitative or quantitative associations between climate and diseases. Synthesising data from literature is particularly useful when primary data is inadequate for the task. However, this approach has limitations, such as difficulty in finding information directly relevant to specific research questions, geographical publication bias, overrepresenting temperate, high‐income countries and underrepresenting tropical regions including Asia (Van de Vuurst and Escobar 2023). There are challenges in extrapolating data from particular ecosystems, populations, agricultural and cultural practices and applying the findings to different environments.

Experimental studies, whether they are laboratory‐based or field‐based, have long contributed to this body of knowledge (N. R. Council 2001). These studies can establish precise associations between climatic factors and pathogens or vectors, such as optimal and suboptimal survival conditions. For instance, studies have examined the growth rate of *Salmonella* under various isothermal conditions (Juneja et al. 2007; Velugoti et al. 2011), or thermal preferences of malaria mosquito *Anopheles* spp. (Shocket et al. 2024; Blanford et al. 2009). While these studies provide precise information, they are usually expensive and cannot address diverse conditions or complex, incompletely understood scenarios.

Mechanistic models predict disease incidence or vector distributions based on known covariates and processes (Sadeghieh et al. 2020). These include degree‐day models, which predict the growth of organisms based on the accumulated thermal energy (Moore et al. 2012; Iwamura et al. 2020). These models are useful for predicting disease incidence or vector distributions under specific conditions. However, they have limited generalisability across ecosystems, limited accountability of external factors and are unable to address incompletely defined relationships among climate variables.

Statistical analyses of observational data play a key role in identifying disease risk factors, including climatic and environmental variables (Haque et al. 2024; Metcalf et al. 2017). While climate data sets are often available at high resolution with minimum bias, biological data are often limited in quality and coverage to address climate change, due to resource constraints and challenges in epidemiological characterisation. The quality of these studies relies on study designs, robust a priori hypotheses and the availability of intermediate data, such as wildlife, vector and environmental data. Traditional regression models may struggle to deal with collinearity among variables and with nonlinear relationships, common in these environmental data (Metcalf et al. 2017). These constraints make it challenging to analyse data with these traditional approaches without precise knowledge of disease ecology or a lack of high‐quality data.

There are newer analytical methods now available which deal better with interpreting complex evidence of interactions between climate and disease.

Machine learning approaches have emerged as powerful tools for analysing complex data sets without requiring extensive prior knowledge of disease ecology. These data‐driven methods are increasingly used in climate‐related epidemiological studies. For instance, ecological niche modelling predicts species occurrence based on environmental variables (Lawrence et al. 2023), including the maximum entropy (MaxEnt) algorithm (Shirzad et al. 2023). Various machine learning algorithms, such as random forest, support vector machine or more advanced algorithms such as deep learning and artificial neural networks, are effectively used for predicting disease occurrence under given climate change scenarios (Zhao et al. 2020; Nguyen et al. 2022). These algorithms excel at processing and uncovering hidden patterns in complex data sets and are considered highly accurate. However, these models have limitations; they do not establish causation and are highly dependent on the quality and structure of input data, making them vulnerable to biases if the data are flawed or poorly selected. In addition, more advanced deep learning models lack interpretability, limiting their ability to provide meaningful insights into underlying mechanisms. Thorough validation and testing are essential to ensure the reliability of these models.

Emerging research is focused on developing interpretable machine learning models to address these gaps, making models more transparent, meaningful and accountable (Murdoch et al. 2019).

## 7 Adaptation of Diseases to Climate Change

### 7.1 Evolution of Known Diseases

It must be expected that as climate change, human behaviour and environmental management practices jointly modify a range of components of the climatic and physical environment, pathogens and vectors will evolve to take advantage of new opportunities or respond to unfavourable trends.

Table 2 summarises typical features of diseases in each of the nine epitypes. Diseases all have individual epidemiological characteristics, so there are exceptions to the characteristics listed; but the purpose of the table is to provide a basis for outlining the differences between epitypes in how they are expected to respond to climate change.

**TABLE 2:** Major characteristics of nine epitypes of diseases, likely effects of climate and risk of disease emergence and evolution.

Item	Epitype 1	Epitype 2	Epitype 3	Epitype 4	Epitype 5	Epitype 6	Epitype 7	Epitype 8	Epitype 9
Transmission	Direct	Direct	Fomites	Direct	Indirect	Indirect	Indirect	Indirect	Direct
Maintenance	Host	Host	Host	Host/environment	Host	Two hosts	Two hosts	Two hosts	Environment
Vector	No	No	No	No	Yes	Yes	Yes	Yes	No
Second host process required	No	No	No	No	No	No	Yes	Yes	No
Second host type	No	No	No	No	Invertebrate	Invertebrate	Vertebrate	Invertebrate	No
Temperature effect	Mild	High	High	High	Moderate	High	Moderate	High	Moderate
Precipitation effect	Mild	High	High	High	Moderate	High	Moderate	High	High
Extreme weather events	No	Moderate	Moderate	High	Moderate	High	Moderate	High	Moderate
Geographical range change	Slow	Rapid	Slow	Slow	Slow	Rapid	Moderate	Moderate	Limited
Climate effect on importance	Low	High	Mild	Moderate	Mild	High	Moderate	Moderate	Low
Response feasible	Moderate	High	High	Moderate	Mild	Moderate	Mild	Moderate	Mild
Risk of new diseases emerging	Low	High	Moderate	Moderate	Low	High	Mild	Moderate	Low
Risk of diseases evolving	Low	High	Moderate	High	Moderate	High	Moderate	High	Moderate

Epitypes 1 and 9 are expected to show the least substantial response and the lowest probability of new diseases emerging within these epitypes. The detailed discussion of individual diseases within each epitype shows that some change will occur, but it will be limited in nature and is expected to largely involve changes in incidence, in part as a consequence of changes in size and nature of populations at risk, not the evolution of the diseases to develop novel behaviours.

Epitype 3 diseases are dependent on survival on fomites, and the time for which they remain infectious in the environment will depend on the thermal response of each organism to rising temperatures, as previously discussed, plus changes in the size of particular populations at risk. The scale of change in incidence is expected to be modest.

Changes in Epitype 5 diseases will depend on changes in the size and geographical distribution of the mechanical vectors. Screw worm flies are the most significant members of this epitype and are likely to expand in distribution and local population sizes as temperatures increase, spreading to new areas and causing more human infections. Other members of this epitype typically fit into other epitypes as well, so the response to climate change will be largely determined by their other transmission mechanisms.

Epitype 7 disease can only occur in locations where both definitive and intermediate hosts exist. It is likely that these diseases will expand in geographical range and incidence, but the changes will be by gradual accretion of new endemic areas rather than by rapid extension into new localities distant from their current range. They are unlikely to evolve to new epidemiological forms.

Epitype 8 diseases share with Epitype 7 the requirement that both definitive and intermediate hosts must be present for the disease to occur. Diseases in this epitype which have flying insects as their intermediate hosts will expand rapidly into new areas as meteorological conditions allow them to maintain populations there, since the definitive hosts are typically already present. Diseases which have nonflying arthropod intermediate hosts might seem unlikely to spread rapidly to new areas and be limited to expansion by accretion. However, because they rely on attachment to hosts for feeding, they can easily move with definitive hosts such as livestock, wild mammals and birds, and moves may occur over long distances with establishment of new remote populations if the sites where they arrive meet their requirements for survival and breeding. Epitype 8 members which rely on intermediate hosts with restricted mobility, such as snails, will expand only by accretion but may benefit from events such as widespread flooding.

Epitype 4 consists of diseases transmitted by the oral route—by deliberate consumption. Most members of this epitype are already distributed very widely in the world, so the main effect of climate change will be to increase incidence, since warmer temperatures favour maintenance in the environment and transmission of these organisms.

Epitype 6 is one of the two epitypes expected to increase greatly in importance as a result of climate warming and other meteorological developments. The most rapid and extensive geographical expansions will occur with organisms transmitted by flying insects. There are many viruses which qualify, and although some already have large ranges, others are limited in distribution by the geographical extent of the ranges of the reservoir and amplifying hosts, and the insects capable of transmitting the agents. Importantly, many of these organisms can be transmitted by multiple species of insects, each of which has a different geographical range, which is likely to change as a result of changes in temperature and precipitation. Unusual weather events may also result in the movement of insects over very large distances, potentially establishing viral populations in new areas. Based on experience with Japanese encephalitis, there may also be a shift in the relative importance of different virus strains, as in the case of a previously rare strain from Indonesia spreading across Australia over a few months and becoming endemic.

While nonflying arthropods do not have the same opportunity to take advantage of meteorological events, if their hosts are mobile they can be transported to new locations, sometimes very long distances away. The main tick vector of Crimean Congo Haemorrhagic Fever is *Hyalomma marginatum* , which is a common ectoparasite of passerine birds and can be carried long distances to potential new establishment sites (Estrada‐Pena et al. 2021; Jameson et al. 2012). It can also be carried on livestock when they are transported by people. Whether it establishes long term depends on the environment at the arrival site (Sajid et al. 2018). New tick‐borne zoonoses continue to be identified in Asia (Wang, Wei, et al. 2021) and have the potential to spread to additional areas under the influence of climate change.

Epitype 2 contains the largest range of organisms capable of increasing their geographical range and prevalence and is also most likely to be the source of new pathogens which have the potential to achieve epidemic or pandemic spread. This is because spread occurs by the respiratory route or by direct contact between source host and susceptible recipient. Among the organisms in this epitype are some with a high capacity to evolve and adapt to new host species, as occurs with avian influenza viruses, due to the fact that they are segmented RNA viruses, with constant production of new variants with different epidemiological characteristics. The organisms which are likely to emerge will probably come mainly from reservoir host species with which people have had limited contact in the past, because otherwise these pathogens would already be in the human population. Bridge hosts play an important role in transferring infection from these reservoir hosts to people. A second transfer facilitating factor is greater direct contact between people and infected species, as human population density and mobility bring people into closer contact with wildlife (Lee et al. 2014).

### 7.2 Emergence of New Diseases

By definition, emerging zoonoses cannot be characterised until they have emerged and developed identifiable epidemiological characteristics in the human population. Emergence can be defined in various ways, but the most practical definition for a zoonotic organism is that it has emerged when the first evidence of human infection occurs; since at that point it becomes a zoonosis.

Some zoonoses appear to emerge quite rapidly, as in the case of SARS (Xu et al. 2004) and SARS‐CoV‐2 (Keusch et al. 2022). Bridge hosts may contribute to rapid emergence, and in some instances the starting point (index case and occasionally also the primary case) can be identified, either at the time or at a later point when sufficient evidence becomes available. Others follow a slower and less clearly defined process of adaptation to new species, including people, over years (Farag et al. 2018; Sikkema et al. 2019), decades (Sharp and Hahn 2011) or even centuries (Jesse et al. 2022). There may also be stages in the emergence process, as in the case of avian influenza viruses, which typically emerged into a pathogen of one additional species, usually the pig, and later extended to other species (Ma et al. 2009). However, the H5N1 avian virus which emerged in the 1990s bypassed the intermediate host stage to directly cause human infections (Kocer et al. 2013). Thus, emergence is a complex and typically a multistage process which may be influenced at various points by climatic factors.

There will be primary and secondary effects of climate change on disease emergence. Primary effects are a direct consequence of changes in temperature and the hydrological cycle (Mora et al. 2022; El‐Sayed and Kamel 2020). These may be quite varied in the effects that occur and their impact on different epitypes of organisms, as described earlier. Effects of temperature may be more than just the consequences of a new level, but also the way in which temperature varies within days and over longer time periods (Rohr and Cohen 2020). Changes in the hydrological cycle may directly affect the transmission of organisms through water (Nichols et al. 2018), but also affect humidity, with consequences for airborne pathogen transmission and extreme weather events, which may influence pathogens as described earlier and facilitate disease emergence.

Secondary effects are consequences of the primary effects, and include changes in biodiversity and ecosystem structure, land use, distribution of wild and domestic animals, human behaviour and population distribution, Efforts are being made to use this evidence to identify areas at high risk of emergence of novel pathogens, mainly viruses (Muylaert et al. 2022, 2023), and to predict the interacting effects of influential factors on emergence of new zoonoses (Becker et al. 2022; Carlson et al. 2021). A global analysis to identify how climate change and consequent changes in land use will increase the risk of transmission of viruses between species (Carlson et al. 2022) identified Asia as one of the two highest risk areas for this to occur. It evaluated the extent of future first interactions between species that could result in transfers, the species expected to make the greatest contribution to transfers, and the effect of human population size and structure. The cumulative number of risky encounters will continue to rise over the time to 2110.

## 8 Response of Disease Surveillance and Control Strategies to Climate Change

**FIGURE 1:**
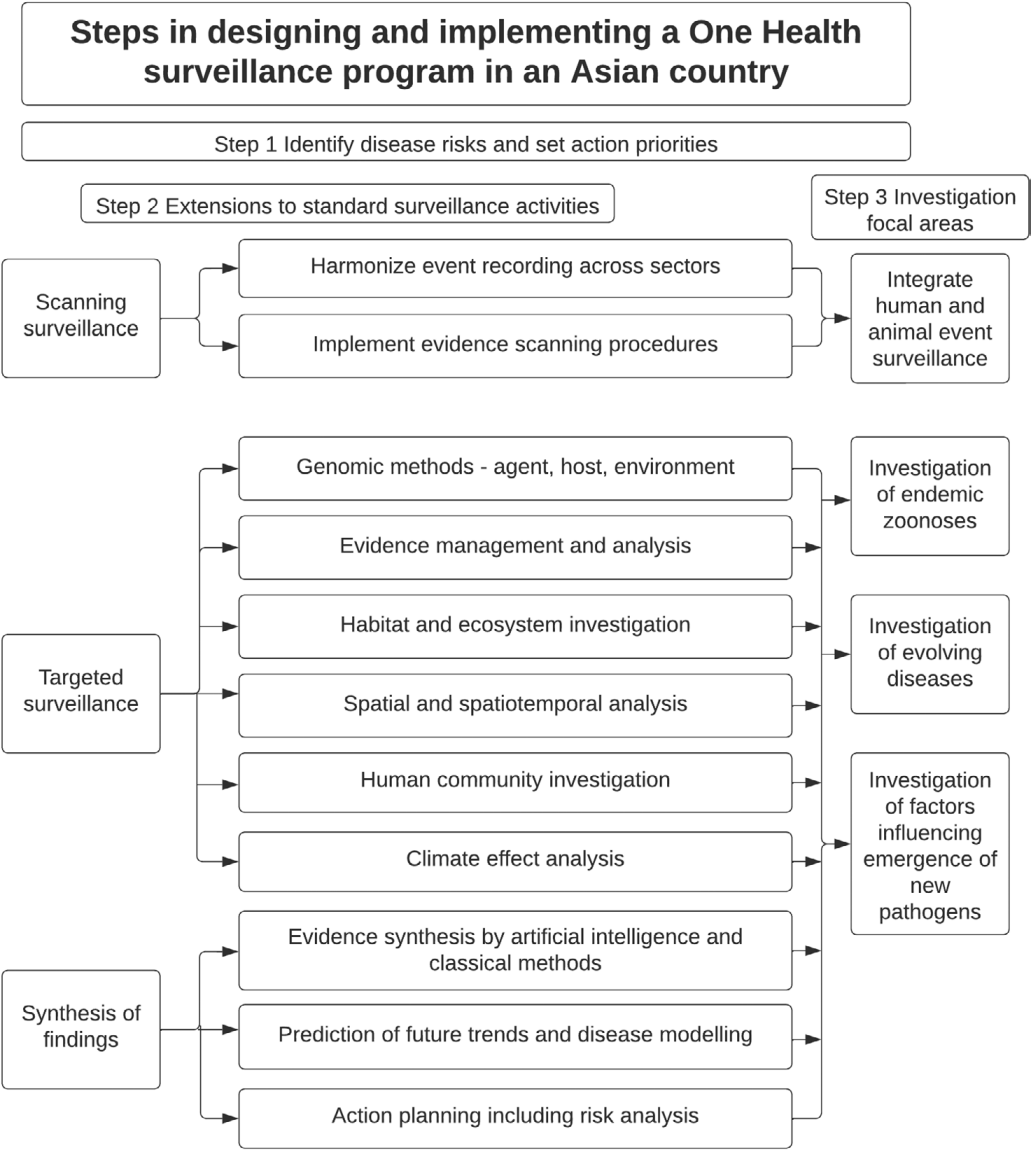
Design of a three‐step plan for implementing One Health surveillance in an Asian country.

A strategy for providing surveillance and response strategies in Asia appropriate for the next decade has been covered in detail in a related paper (Morris and Wang 2024). A “One Health” strategy is required, in which human surveillance, animal surveillance and other investigational methods are combined in an integrated approach. The steps are summarised in Figure 1.

Analysis of climate influences on zoonotic disease emergence and evolution is a core part of the strategy. Development of an approach for Asia will be much more effective if countries in the region coordinate their efforts in order to consider region‐wide effects rather than purely national consequences of climate change, since the expected trends will not respect national borders and may produce region‐wide disease events. Endemic zoonoses in Epitypes 2 and 6 that are considered high risk for evolving under the influence of climate change can be investigated using targeted surveillance strategies to build understanding of their current and expected future epidemiological behaviour.

This should be conducted in areas which have been identified in spatial predictions as high risk for occurrence of disease emergence or evolution, since these typically have multiple risk factors operating. If possible, evidence from these locations should be compared with evidence from areas considered low risk to test the hypothesis that prediction of risk locations is feasible. These investigations will allow investigators to gain expertise they can use in the event of a possible emerging disease being identified.

## 9 Conclusion

It is clear that substantial changes in spatiotemporal patterns of zoonoses will occur as a consequence of predicted changes in the Asian climate. These changes will vary considerably among different categories of diseases and to facilitate the evaluation of the various categories, a classification system into nine epitypes has been developed, which can be used for both animal diseases and zoonoses. It uses transmission method and maintenance method in populations and environments to group diseases.

The expected changes for each epitype are summarised in Table 2.

Epitypes 2 and 6 are those expected to create the greatest future problems as a result of the influence of climate change. This is expected to be mediated by both increased incidence in endemic areas and range extension to new areas. However, while Epitype 2 diseases will disseminate by direct transmission between hosts, Epitype 6 diseases will be dependent on the movement of their vector species.

Epitype 4 will increase in importance, but range extension will not be as significant, since these pathogens are already very widely distributed. Epitypes 7 and 8 will change mainly by range extension, typically by accretion, but in some cases by longer distance movements. Epitypes 3 and 5 are expected to show moderate increases in importance, while Epitypes 1 and 9 will show only limited change. Across the full range of zoonoses, there will be exceptions to typical patterns in some respects, and this has been demonstrated using the example zoonoses for each epitype. The description in Table 2 is, however, expected to apply to most diseases in each epitype.

New zoonoses are being regularly identified, as contacts between wildlife and people increase, both directly and indirectly through bridge hosts or vectors. Experience with recent zoonoses such as the three SARS coronaviruses and avian influenza H5N1 shows the potential for future zoonotic diseases to cause severe social and economic disruption.

If we are to mitigate the risks of future emerging or evolving zoonotic pathogens causing regional epidemics or global pandemics, it is essential that there be investment in surveillance activities and response capability development for Asia, one of the main sources of risk pathogens. This will require an integrated approach, with countries cooperating to build understanding and capability.

## Conflicts of Interest

The authors declare no conflicts of interest.

## Supporting information


**Data S1:** zph70007‐sup‐0001‐Supinfo.docx.

## Data Availability

Details of epitypes for the 161 zoonoses and animal diseases used to create Table 1 are given in Supporting Information. No other original data were created for this paper.
