# Supplementary material for: The Effect of Climate Change on Emergence and Evolution of Zoonotic Diseases in Asia
Source: Zoonoses Public Health. 2025 Sep 1;72(7):587–611. doi: 10.1111/zph.70007 (PMC12508791; doi:10.1111/zph.70007)
Supplement: Supplementary file 1 — Data S1: zph70007‐sup‐0001‐Supinfo.docx. [file ZPH-72-587-s001.docx]

**Supplementary Material**

**The Effect of Climate Change on Emergence and Evolution of Zoonotic Diseases in Asia**

**Roger S. Morris and Masako Wada**

This list provides the epitypes which were used to create Table1. It shows epitypes for diseases when transmitted between animals, and epitypes for transmission of zoonoses between animals and people. In total 161 diseases are listed. The 120 diseases of mammals and birds are taken from the list of diseases reported biannually to the World Organization for Animal Health. Some are zoonoses. An additional 41 zoonoses are derived from lists provided by the Centers for Disease Control and Prevention in the United States, <https://www.cdc.gov/healthy-pets/diseases/index.html#cdc_facts_stats_trends-a-to-z-list-of-zoonotic-diseases>

United Kingdom list <https://www.gov.uk/government/publications/list-of-zoonotic-diseases/list-of-zoonotic-diseases>

and zoonoses considered in this paper. Because some diseases fit more than one epitype due to two or three separate transmission methods, there are 177 epitypes listed for the 161 diseases in animals. There are 101 epitypes describing transmission methods to people for 87 zoonotic diseases. The list of zoonoses is far from comprehensive, but intended to provide examples of zoonoses recognized as important.

The objective of producing the list was to indicate the distribution of animal diseases and zoonoses between epitypes, not to definitively report the epitypes of individual diseases. The epidemiological situation for some diseases is complex, or incompletely determined, and the list is a brief statement of the authors’ assessment of appropriate epitypes, which is open to further deliberation.

| **Epitype** | **Species mainly affected** | **Zoonotic epitype** | **Disease** |
| --- | --- | --- | --- |
|  |  |  | **WOAH List** |
| 9 | Multiple |  | Aeromonas hydrophila (Inf. with) |
| 8 | Equids |  | African horse sickness virus (Inf. with) |
| 2 | Suids |  | African swine fever virus (Inf. with) |
| 9 | Multiple | 3 | Anthrax |
| 2 | Pig |  | Atrophic rhinitis of swine (-2005) |
| 2 | Multiple |  | Aujeszky's disease virus (Inf. with) |
| 2 | Multiple | 2 | Avian chlamydiosis |
| 2 | Poultry |  | Avian infectious bronchitis |
| 2 | Poultry |  | Avian infectious laryngotracheitis |
| 2 | Poultry |  | Avian mycoplasmosis (M.synoviae) (2006-) |
| 2 | Poultry |  | Avian mycoplasmosis (Mycoplasma gallisepticum) |
| 3 | Poultry |  | Avian tuberculosis (-2005) |
| 8 | Ruminants |  | Bluetongue virus (Inf. with) |
| 9 | Multiple | 4 | Botulism (-2014) |
| 8 | Cattle |  | Bovine anaplasmosis |
| 8 | Cattle |  | Bovine babesiosis |
| 7 | Cattle | 7 | Bovine cysticercosis (-2014) |
| 1 | Cattle |  | Bovine genital campylobacteriosis |
| 4 | Cattle | 4 | Bovine spongiform encephalopathy |
| 2 | Multiple | 2 | Bovine tuberculosis (-2018) |
| 2 | Cattle |  | Bovine viral diarrhoea (2006-) |
| 2 | Cattle | 3,4 | Brucella abortus (Inf. with) |
| 2 | Sheep, goat | 3,4 | Brucella melitensis (Inf. with) |
| 2 | Suids | 3,4 | Brucella suis (Inf. with) |
| 2 | Equids | 2 | Burkholderia mallei (Inf. with) (Glanders) |
| 2 | Camels |  | Camelpox (2006-) |
| 2 | Goat |  | Caprine arthritis/encephalitis |
| 2 | Sheep, goat | 3 | Chlamydia abortus (Inf. with) (Enzootic abortion of ewes, ovine chlamydiosis) |
| 2 | Pig |  | Classical swine fever virus (Inf. with) |
| 2 | Sheep, goat |  | Contagious agalactia |
| 2 | Goat |  | Contagious caprine pleuropneumonia |
| 1 | Horse |  | Contagious equine metritis |
| 6 | Multiple | 3,6 | Crimean Congo haemorrhagic fever (2006-) |
| 3 | Multiple | 3 | Dermatophilosis (-2005) |
| 1 | Equids |  | Dourine |
| 2 | Ducks |  | Duck virus enteritis (-2005) |
| 2 | Ducks |  | Duck virus hepatitis |
| 7 | Multiple | 7 | Echinococcosis/hydatidosis |
| 7 | Multiple | 7 | Echinococcus granulosus (Inf. with) (2014-) |
| 7 | Multiple | 7 | Echinococcus multilocularis (Inf. with) (2014-) |
| 6 | Dog |  | Ehrlichia canis (Inf. with) |
| 2 | Pig |  | Enterovirus encephalomyelitis (-2005) |
| 1 | Cattle |  | Enzootic bovine leukosis |
| 6 | Ruminants |  | Epizootic hemorrhagic disease virus (Inf. with) |
| 9 | Equids |  | Epizootic lymphangitis (-2005) |
| 2 | Equids |  | Equid herpesvirus-1 (Inf. with) (Equine rhinopneumonitis) (2014-) |
| 1,2 | Equids |  | Equine arteritis virus (Inf. with) |
| 6 | Equids | 6 | Equine encephalomyelitis (Eastern and Western)(-2005) |
| 6 | Equids | 6 | Equine encephalomyelitis (Eastern)(2006-) |
| 6 | Equids | 6 | Equine encephalomyelitis (Western)(2006-) |
| 6 | Equids |  | Equine encephalosis virus (Inf. with) |
| 5,1,3 | Equids |  | Equine infectious anaemia |
| 2 | Equids |  | Equine influenza virus (Inf. with) |
| 6 | Equids |  | Equine piroplasmosis |
| 2 | Equids |  | Equine rhinopneumonitis (-2013) |
| 2,3 | Multiple |  | Foot and mouth disease virus (Inf. with) |
| 2,3 | Poultry | 2,3 | Fowl cholera (-2011) |
| 2,3,6 | Poultry |  | Fowl pox (-2005) |
| 2,6,1 | Poultry |  | Fowl typhoid |
| 3 | Bovids |  | Haemorrhagic septicaemia |
| 6 | Ruminants |  | Heartwater |
| 2 | Horse | 2 | Hendra viruses (Inf. with) |
| 2 | Poultry | 2 | Highly pathogenic avian influenza (poultry) |
| 2 | Birds | 2 | Highly pathogenic influenza A viruses (Inf. with)(non-poultry including wild birds)(2017-) |
| 2 | Horse |  | Horse mange (-2005) |
| 2 | Horse |  | Horse pox (-2005) |
| 2 | Bovids |  | Infectious bovine rhinotracheitis/infectious pustular vulvovaginitis |
| 3 | Poultry |  | Infectious bursal disease (Gumboro disease) |
| 2 | Multiple | 2 | Influenza A virus (Inf. with) |
| 6 | Multiple | 6 | Japanese encephalitis |
| 8 | Multiple | 8 | Leishmaniosis |
| 2 | Multiple | 2 | Leptospirosis |
| 2 | Multiple | 2 | Low pathogenic avian influenza (poultry)(2006-) |
| 6 | Bovids |  | Lumpy skin disease virus (Inf. with) |
| 2 | Sheep, goat |  | Maedi-visna |
| 2 | Cattle |  | Malignant catarrhal fever (wildebeest only)(2006-2008) |
| 2 | Poultry |  | Marek's disease (-2011) |
| 2 | Camels | 2 | Middle East respiratory syndrome coronavirus (MERS-CoV)(Inf. with) |
| 2 | Monkeys | 1,2 | Mpox |
| 2 | Multiple | 2 | Mycobacterium tuberculosis (Inf. with)(-2017) |
| 2 | Multiple | 2 | Mycobacterium tuberculosis complex (Inf. with)(2019-) |
| 2 | Bovids |  | Mycoplasma mycoides subsp. mycoides SC (Inf. with) (Contagious bovine pleuropneumonia) |
| 6,2 | Rabbits |  | Myxomatosis |
| 6 | Sheep, goat | 6 | Nairobi sheep disease |
| 5 | Multiple | 5 | New world screwworm (Cochliomyia hominivorax) |
| 2 | Poultry |  | Newcastle disease virus (Inf. with) |
| 5 | Multiple | 5 | Old world screwworm (Chrysomya bezziana) |
| 1 | Sheep, deer |  | Ovine epididymitis (Brucella ovis) |
| 2 | Sheep, goat |  | Ovine pulmonary adenomatosis (-2005) |
| 2 | Ruminants |  | Paratuberculosis |
| 2 | Sheep, goat |  | Peste des petits ruminants virus (Inf. with) |
| 2 | Birds |  | Pigeon rotavirus |
| 2 | Pig |  | Porcine epidemic diarrhoea virus (Inf. with) |
| 2 | Pig |  | Porcine reproductive and respiratory syndrome virus (Inf. with) |
| 2 | Pig |  | Postweaning Multisystemic Wasting Syndrome (PMWS) |
| 2 | Multiple |  | Pox viruses (other than those listed by the OIE)(Inf. with)(2011-) |
| 1,2 | Poultry |  | Pullorum disease |
| 2 | Ruminants | 2 | Q fever |
| 2,5 | Rabbits |  | Rabbit haemorrhagic disease |
| 1 | Multiple | 1 | Rabies virus (Inf. with) |
| 6 | Multiple | 6 | Rift Valley fever virus (Inf. with) |
| 2 | Sheep |  | Salmonellosis (S. abortusovis) |
| 2 | Multiple | 2 | SARS-CoV-2 in animals (Inf. with) |
| 6 | Ruminants |  | Schmallenberg virus (Inf. with) |
| 2 | Sheep, goat |  | Scrapie |
| 2 | Sheep, goat |  | Sheep pox and goat pox |
| 2 | Pig | 2 | Streptococcus suis (Inf. with) |
| 6 | Multiple |  | Surra (Trypanosoma evansi) |
| 2 | Pig |  | Swine vesicular disease (-2014) |
| 6 | Pig | 6 | Taenia solium (Inf. with) (Porcine cysticercosis) |
| 6 | Ruminants |  | Theileriosis |
| 2 | Pig |  | Transmissible gastroenteritis |
| 1 | Pig | 4 | Trichinella spp. (Inf. with) |
| 1 | Bovids |  | Trichomonosis |
| 6 | Bovids | 6 | Trypanosomosis (tsetse-transmitted) |
| 6 | Multiple | 6 | Tularemia |
| 2 | Turkey |  | Turkey rhinotracheitis (2006-) |
| 6 | Equids | 6 | Venezuelan equine encephalomyelitis |
| 2 | Pig |  | Vesicular stomatitis (-2014) |
| 6 | Multiple | 6 | West Nile Fever |
|  |  |  | **Additional zoonoses, mainly from US CDC and UK lists, not included above** |
| 2,3 | Dog, cat | 2,3 | Ancylostoma brazilienze |
| 2,3 | Dog, cat | 2,3 | Ancylostoma caninum |
| 2 | Cat | 1 | Bartonella henselae |
| 2 | Monkeys | 2 | B virus (Herpes B) |
| 6 | Multiple | 6 | Borrelia burgdorferi |
| 9 | Multiple | 9 | Cyptococcus neoformans |
| 2 | Cat, dog | 1 | Capnocytophaga spp. |
| 2 | Birds | 2 | Chlamydia psittaci |
| 9 | Multiple | 9 | Clostridioides difficile |
| 2 | Multiple | 2 | Contagious ecthyma (parapox) |
| 2 | Multiple | 2,4 | Corynebacterium ulcerans |
| 2,4 | Multiple | 2,4 | Cryptosporidium |
| 2 | Multiple | 2 | Dermatophytes (Microsporum, Trichophyton) |
| 5 | Dog, cat | 5 | Dipylidium |
| 2 | Multiple | 2 | Ebolavirus |
| 2 | Pig, fish | 2 | Erysipelothrix |
| 4 | Multiple | 4 | Escherichia coli (specific strains) |
| 8 | Multiple | 8 | Fascioliasis |
| 4 | Multiple | 4 | Giardia |
| 2 | Rodents | 2 | Hantavirus |
| 4 | Pig | 4 | Hepatitis E |
| 9 | Multiple | 9 | Histoplasma |
| 2,6 | Multiple | 2,6 | Kyasanur Forest Disease |
| 2 | Rodents | 2 | Lassa fever |
| 4 | Multiple | 4 | Listeria monocytogenes |
| 6 | Multiple | 6 | Louping ill |
| 2 | Rodents | 2 | Lymphocytic choriomeningitis |
| 2 | Bats | 2,4 | Nipah virus |
| 6 | Rodents | 6 | Orientia tsutsugamushi (scrub typhus) |
| 2 | Multiple | 2 | Pasteurella spp. |
| 1 | Rodents | 1 | Rat bite fever (Streptobacillus moniliformis, Spirillum minus) |
| 6 | Multiple | 6 | Rickettsia rickettsiae |
| 2 | Multiple | 1,2 | Sarcoptes scabiei |
| 2 | Cat | 2 | Sporothrix schenkii (Sporotrichosis) |
| 2 | Horse | 2 | Streptococcus zooepidemicus |
| 4 | Dog, cat | 4 | Toxocariasis |
| 4 | Multiple | 1,4 | Toxoplasma gondii |
| 4 | Canids | 2 | Uncinaria stenocephala |
| 2 | Primates | 2 | Yellow fever virus |
| 4 | Multiple | 4 | Yersinia enterocolitica and Y. pseudotuberculosis |
| 6 | Rodents | 6 | Yersinia pestis |
